# Psychological responses during the COVID-19 outbreak among university students in Bangladesh

**DOI:** 10.1371/journal.pone.0245083

**Published:** 2020-12-31

**Authors:** Md. Saiful Islam, Md. Safaet Hossain Sujan, Rafia Tasnim, Md. Tajuddin Sikder, Marc N. Potenza, Jim van Os

**Affiliations:** 1 Department of Public Health and Informatics, Jahangirnagar University, Savar, Dhaka, Bangladesh; 2 Youth Research Association, Savar, Dhaka, Bangladesh; 3 Department of Psychiatry and Child Study Center, Yale School of Medicine, New Haven, Connecticut, United States of America; 4 Connecticut Mental Health Center, New Haven, Connecticut, United States of America; 5 Connecticut Council on Problem Gambling, Wethersfield, Connecticut, United States of America; 6 Department of Neuroscience, Yale University, New Haven, Connecticut, United States of America; 7 Department of Psychiatry, UMC Utrecht Brain Center, University Medical Center Utrecht, Utrecht University, Utrecht, The Netherlands; 8 Department of Psychosis Studies, Institute of Psychiatry, Psychology & Neuroscience, King’s College London, London, United Kingdom; Universitat de Valencia, SPAIN

## Abstract

Mental health problems in students are considered a public health challenge. We assessed the prevalence of depression, anxiety, and stress (DAS) with the DASS-21, as well as associated factors, among university students in Bangladesh early in the COVID-19 outbreak. We hypothesized high levels of DAS and their associations with previously reported factors (e.g., poor sleep, lack of exercise, heavy internet use) and those linked to disadvantage (e.g., low monthly family income). We also enquired about participants’ satisfaction with their pursuit of their academic studies while living under COVID-19 restrictions. An internet-based survey was conducted during the month of April 2020, involving 3,122 Bangladeshi university students aged 18 to 29 years (59.5% males; mean age 21.4±2 years). Prevalence estimates of depression, anxiety and stress were, respectively, 76.1%, 71.5% and 70.1% for at least mild symptoms, 62.9%, 63.6% and 58.6% for at least moderate symptoms, 35.2%, 40.3%, and 37.7% for at least severe symptoms and 19.7%, 27.5% and 16.5% for at least very severe symptoms. The present estimates of DAS were more prevalent than in previous pre-COVID-19 studies among Bangladeshi university students. Regression analyses with DASS-21-score as a dependent variable revealed associations with factors mostly as hypothesized. The largest effect size on DAS symptoms was related to students’ satisfaction with their academic studies during the pandemic. As this survey used cross-sectional and self-reported methods, causality cannot be inferred. Mental health monitoring of students attempting to cope with the impacts of the COVID-19 outbreak may be useful and feasible.

## Introduction

Many jurisdictions have been impacted by the COVID-19 (Coronavirus disease 2019) pandemic. COVID-19, caused by the SARS-CoV-2 virus, is a highly contagious respiratory disease spread through droplets from infected persons who may be symptomatic or asymptomatic [1]. The virus, first identified in Wuhan, China, in December 2019, has affected 215 countries, areas, or territories with 230,104 deaths and 3,272,202 positive cases globally, as of May 03, 2020 [2]. On March 11, 2020, the World Health Organization declared a pandemic [3], and the first three cases of COVID-19 were found in Dhaka, Bangladesh on March 08, 2020 [4–6]. Given the dense population of Bangladesh and other factors, COVID-19 has become a major public health concern in Bangladesh, as in other countries. According to the Institute of Epidemiology Disease Control and Research, there were through May 03, 2020, a total of 8,790 Bangladeshi cases confirmed, which included 175 people who had died and 177 people had who recovered from COVID-19 [7].

To reduce the spread of COVID-19, a public holiday was declared by the Government of Bangladesh on March 26, 2020 [8]. All schools, colleges, and universities have remained closed since. This situation may hinder university students’ studies, disrupt their daily routines and habits, and impact their mental health. Further, home quarantine, physical/spatial distancing, and other restrictions are likely to have psychological impacts on students [9] and negatively influence their mental wellbeing [10].

Public health crises can elicit considerable negative emotions, in line with stress [11] and perceived risk [12] theories. For students, COVID-19-related stressors may include health concerns emanating from increases in cases, sequelae of distancing/isolation strategies, and disruptions in starting classes and taking exams [13]. These may lead to feelings of hopelessness, fear of death, and frustration that may grow among students in quarantine [14]. Furthermore, the situation’s unpredictability and volatility about when and how to manage the disease and reduce risk may be particularly challenging and demanding [14].

While quarantined and out of the university environment and schedule, students may experience stress, anxiety, anger, boredom, loneliness, and other emotions, with both shorter- and longer-term impacts [15–17]. In the shorter-term, such feelings may lead to sleep problems, changes in eating habits, and engagement in potentially addictive behaviors, and some of these factors may then also increase DAS [18]. News, misinformation, and rumors about COVID-19 may also increase negative thoughts and emotions [19] within students and about their futures.

Quarantine measures once unprecedented have now become widespread [15] and may contribute to poorer mental states in students. However, little information exists on the mental status of students during the COVID-19 pandemic. Obtaining structured measures of DAS may help assess the need for interventions to minimize mental health impacts of the pandemic on students. A prior study reported high levels of moderate to extremely severe depression (52.2%), anxiety (58.1%), and stress (24.9%) during pre-COVID-19 periods among university students in Bangladesh [20]. Several factors leading to DAS among students have been established in existing literature, including sex, strained relationships, family and peer pressure, lack of financial support and hardship, high parental expectations, sleep deprivation, problematic internet use, longer screen time, isolation, toxic psychological environment, academic pressure, workload, and heavy test schedules [20–26]. We aimed to replicate and extend these findings in Bangladeshi university students, focusing on levels of DAS and factors associated with these measures, in April 2020. We hypothesized that there would be high levels of DAS. Further, we hypothesized that these would be associated with factors previously linked to depression and anxiety in this population (lower socioeconomic status, lack of exercise, lack of sleep, smoking, heavy internet use and female gender [20, 21, 27]). In addition, we hypothesized that the following variables would additionally be associated with poorer mental health under the circumstances of the lockdown: urban residence, given that crowded cities arguably would represent a worse environment when under lockdown; single marital status, given lower levels of support in exceptional circumstances; lower age, given less experience of living independently away from home; being from a large family and from an extended family, as these groups may have limited financial resources as more individuals are dependent on the main household income which does not increase linearly with number of dependents [28]. In addition, we enquired about the impact of the present circumstances on study satisfaction, hypothesizing that more negative impact on study satisfaction would be associated with poorer mental health.

## Methods

### Study procedures

This study employed a cross-sectional design and surveyed 3,122 individuals recruited from Bangladeshi university students during the month of April 2020. Students from public, private, and national universities in Bangladesh participated in the survey. The survey targeted active (current) students. Participants were recruited using convenience sampling through an online survey. The questionnaire was translated in Bangla (the native language of participants) and then translated back to English by different experts (with very good command over both the Bangla and English languages). A pilot test was conducted with 40 individuals from the same student population to assess the questionnaire in terms of acceptability and clarity. Minor adjustments were incorporated after the pilot. These questionnaires were excluded from the final analysis. The survey was conducted with an online survey tool (Google Forms). No incentives or rewards were offered for participation. During the survey, individuals first provided informed consent and then were asked the following question: “*Are you willing to participate in this study freely and voluntarily*?”. If the person answered ‘no’, a blank survey form was submitted automatically. If the person answered ‘yes’, he/she was given access to the full survey form. In order to collect the convenience sample, the link of the online survey was shared across different online platforms (e.g., Facebook, WhatsApp, student blogs, etc.) to obtain sufficient responses. Initially, 3,216 respondents submitted the survey form after providing informed consent. Of these, 3,145 respondents (97.8%) completed the entire survey, voluntarily without any incentives. After removing the surveys that were submitted but missing data, 3,122 were included in the final analysis. The participants consisted of 59.5% males and 40.5% females, and their mean age was 21.4 years (SD = 2) ranging from 18 to 29 years. The inclusion criteria to participate in the study were being a Bangladeshi university student, having internet access, being willing to respond voluntarily and submit a completed survey.

### Ethical considerations

The study was conducted in accordance with the Institutional Research Ethics and Helsinki declaration. All procedures were approved by Biosafety, Biosecurity, and Ethical Clearance Committee, the ethical review board of the Faculty of Biological Sciences, Jahangirnagar University, Savar, Dhaka-1342, Bangladesh. The survey data were collected anonymously, and all participants gave their written informed consent to participate. The consent form clearly documented the (i) nature and procedure of the study, (ii) aims of the study, (iii) anonymity and confidentiality of data, (iv) choice to participate in the study, and (v) right to revoke data at any time from the study.

### Measures

A structured and self-related survey questionnaire containing informed consent, questions regarding socio-demographics, and personal lifestyle-related measures during COVID-19, as well as a psychometric scale (DASS-21) to assess DAS, was used to collect data. In the informed consent, a specific mention was made of the COVID-19 situation and our interest to assess mental health in relation to the COVID-19 outbreak and the ensuing public health measures. One of the questions was related to this situation, namely the question: *Are you satisfied with your studies under the present circumstances*? This question therefore was used as a marker of COVID-19 impact.

#### Socio-demographic measures

Socio-demographic data were collected during the survey including gender, age, marital status, nature of family (nuclear/joint, with joint being an extended family, often of multiple generations), number of family members, monthly family income, and residence (rural versus urban). Monthly family income was classified into three categories (low-income, middle-income, and high-income) and was based on monthly family income of less than 15,000 Bangladeshi Taka (BDT), 15,000–30,000 BDT, and more than 30,000 BDT, respectively.

#### Personal lifestyle-related measures during COVID-19

Personal lifestyle-related measures were collected by asking questions concerning engaging in physical exercise (yes/no), average hours of sleep, sleep satisfaction (yes/no), average hours of browsing the internet, satisfaction with their pursuit of their academic studies while living under COVID-19 restrictions (yes/no), and cigarette smoking (yes/no) during the COVID-19 outbreak. Sleeping hours were classified into three categories on the basis of average daily sleeping hours (normal [7-9h], less than normal [<7h], or more than normal [>9h]) based on the previous studies [29–32]. Alcohol or drugs are not available in the lockdown situation in Bangladesh, particularly not for students. Therefore, these variables were not included in the survey.

#### Depression, Anxiety and Stress Scale (DASS-21)

The DASS-21 is widely used to measure depression, anxiety, and stress. The DASS-21 item is a modified and shorter version of the original DASS-42 item [33]. This self-reported instrument consists of 21 questions including three subscales: 7 items each for DAS with a four-point Likert scale ranging from 0 (“never”) to 3 (“always”) [33]. Sum scores are calculated by adding the scores on the items per subscale (i.e., depression, anxiety, and stress) and multiplying them by 2. This study used the Bangla version DASS-21 [34] to assess DAS. Predefined thresholds for mild, moderate to severe or extremely severe symptom levels were used to categorize levels of DAS, as follows: normal (depression 0–9, anxiety 0–7, and stress 0–14), mild (depression 10–13, anxiety 8–9, and stress 15–18), moderate (depression 14–20, anxiety 10–14, and stress 19–25), severe (depression 21–27, anxiety 15–19, and stress 26–33) and extremely severe (depression ≥28, anxiety ≥20, and stress ≥34) [34]. In the original Bangla version DASS-21, these resulted in excellent reliability (Cronbach’s alpha for the three subscales: depression [α = 0.99], anxiety [α = 0.96], and stress [α = 0.96]), and overall Cronbach’s alpha for DASS-21 was 0.99 [34]. In the present study, the Cronbach’s alpha for the depression, anxiety, and stress subscales were 0.85, 0.85, and 0.86, respectively, and the overall DASS-21 scale was found to have excellent reliability (Cronbach’s alpha = 0.94).

### Statistical analysis

Data analysis was performed using Microsoft Excel 2019 and IBM SPSS Statistics version 25.0. Microsoft Excel was used for editing, sorting, and coding. Next, the excel file was imported into SPSS software. Descriptive statistics (frequencies, percentages, means, standard deviation) were executed using SPSS software. We present prevalence estimates of DAS using the predefined thresholds defined above, facilitating comparison with previous work using similar measures. In order to statistically evaluate associations with the 13 exposure variables, the approximately normally distributed DASS-21 total score was used as the outcome in a multiple linear regression model. The reason for this was that the three subscales of depression, anxiety and stress were very strongly associated with each other (anxiety-depression: Pearson *r* = 0.74, *p*<0.001; anxiety-stress: Pearson *r* = 0.78, *p*<0.001; depression-stress: Pearson *r* = 0.81, *p*<0.001), the first principal component explained 85% of the variance, with an Eigenvalue of 2.6, and the second component had an Eigenvalue of only 0.26. This justifies analyzing the hypotheses at the level of the total score rather than at the level of the three separate subscales [35]. A multiple linear regression model was fitted with the 13 hypothesized exposure variables entered simultaneously, so as to assess their independent associations with DASS-21 total score. Effect sizes were expressed as standardized regression coefficients from the linear regression model. Using the Stata VIF command, we calculated the variance inflation factor for independent variables in order to examine the possibility of multicollinearity. No variable had a value higher than 4, well below the suggested value of 10 indicating possible multicollinearity [36]. Similarly, we plotted the regression residuals using the Stata QNORM command which did not show any substantial departure from normality. The association of variables was considered statistically significant if the two-sided *p*-value was less than to 0.05. We also describe the findings surviving Bonferroni correction for multiple comparisons.

## Results

Around 60% (59.5%) of the participants were male, and mean age was 21.4 years (SD = 2.0), ranging from 18 to 29 years. Descriptive statistics for all variables are presented in Table 1. Most respondents were single (81.9%), came from urban areas (68.3%), were from high-income families (50.2%), were from nuclear families (81.9%), and had a number of family members below five (71.2%). Additionally, most did not engage in regular physical exercise (55.3%), slept a “normal” amount (7–9 hours) (73.3%), were satisfied with their sleep (77.3%), were not satisfied with their pursuit of their academic studies while living under COVID-19 restrictions (61.9%), and did not smoke cigarettes (81.2%). Many also reported frequent internet use (use more than six hours/day; 33.9%).

**Table 1:** Distribution of demographic characteristics and behavioral factors (N = 3,122).

Variables	n	(%)
**Gender**		
Male	1857	(59.5)
Female	1265	(40.5)
**Age**		
18–24 years	2300	(73.7)
25–29 years	822	(26.3)
**Marital status**		
Single	2557	(81.9)
In a relationship	463	(14.8)
Married	102	(3.3)
**Family type**		
Nuclear	2557	(81.9)
Joint	565	(18.1)
**Number of family members**		
<5	2222	(71.2)
≥5	900	(28.8)
**Monthly family income**		
Low	463	(14.8)
Middle	1091	(34.9)
High	1568	(50.2)
**Residence**		
Rural	990	(31.7)
Urban	2132	(68.3)
**Physical exercise**		
Yes	1395	(44.7)
No	1727	(55.3)
**Sleep status**		
Less than normal	457	(14.6)
Normal (7–9 hours)	2288	(73.3)
More than normal	377	(12.1)
**Sleep satisfaction**		
Yes	2414	(77.3)
No	708	(22.7)
**Internet browsing hours**		
<2 hours	229	(7.3)
2–4 hours	931	(29.8)
5–6 hours	904	(29.0)
>6 hours	1058	(33.9)
**Satisfaction with academic study under present circumstances**		
Yes	1189	(38.1)
No	1933	(61.9)
**Smoking (tobacco)**		
Yes	587	(18.8)
No	2535	(81.2)

### Symptoms at different severity levels

Symptoms at predefined levels are depicted in Table 2. Percentages of people acknowledging symptoms decreased with increasing level of severity, but were relatively high even at the highest severity cut-off, particularly for anxiety (27.5%) and less for depression (19.7%) and stress (16.5%).

**Table 2:** Symptoms at predefined severity thresholds.

Characteristics	Depression		Anxiety		Stress	
	n	(%)	n	(%)	N	(%)
At least mild	2375	(76.1)	2233	(71.5)	2188	(70.1)
At least moderate	1963	(62.9)	1985	(63.6)	1828	(58.6)
At least severe	1099	(35.2)	1259	(40.3)	1177	(37.7)
Extremely severe	616	(19.7)	858	(27.5)	514	(16.5)

### Associations with hypothesized factors

The DASS-21 scores were significantly (*p*<0.05) higher among participants who reported being female, being older (25–29 years), having ≥5 family members, living in urban areas, not engaging in physical exercise, having dissatisfaction with sleep, spending more hours browsing the internet, having dissatisfaction with academic studies under the present COVID-19 circumstances, and smoking (Table 3). Among these, being female, living in an urban area, not engaging in physical exercise, having dissatisfaction with sleep, browsing the internet for many hours a day (5–6, 6 or more), having dissatisfaction with their pursuit of their academic studies while living under COVID-19 restrictions, and smoking withstood Bonferroni correction for multiple comparisons. Significant associations between DASS-21 variables were in hypothesized directions. The largest standardized effect size (0.27) was associated with participants’ dissatisfaction with their pursuit of their academic studies while living under COVID-19 restrictions.

**Table 3:** Associations between 13 hypothesized factors and DASS-21 total symptom score.

Variables	DASS-21 Mean score	(SD)	n	β†	*p*-value
**Gender**					
Male	48.27	(29.07)	1857	*	
Female	58.74	(27.96)	1265	0.20	<0.001
**Age**					
25–29 years	54.06	(29.47)	822	*	
18–24 years	51.96	(28.93)	2300	0.03	0.047
**Marital status**					
Single	51.87	(28.99)	2557	*	
In a relationship	55.64	(29.30)	463	-0.004	0.808
Married	54.45	(29.75)	102	0.008	0.621
**Family type**					
Nuclear	53.00	(28.55)	2557	*	
Joint	50.28	(31.30)	565	-0.016	0.398
**Number of family members**					
<5	52.32	(27.75)	2222	*	
≥5	52.97	(32.13)	900	0.05	0.011
**Monthly family income**					
Low	49.53	(25.77)	463	*	
Middle	51.25	(26.89)	1091	-0.03	0.243
High	54.26	(31.29)	1568	-0.04	0.062
**Residence**					
Rural	45.99	(28.32)	990	*	
Urban	55.54	(28.94)	2132	0.06	<0.001
**Physical exercise**					
Yes	46.03	(27.81)	1395	*	
No	57.73	(29.03)	1727	0.09	<0.001
**Sleep status**					
Normal (7–9 hours)	49.69	(27.07)	2288	*	
More than normal	59.49	(28.55)	377	-0.04	0.085
Less than normal	60.86	(35.93)	457	0.02	0.389
**Sleep satisfaction**					
No	68.69	(30.78)	708	*	
Yes	47.76	(26.77)	2414	-0.18	<0.001
**Internet browsing hours**					
<2 hours	42.46	(27.64)	229	*	
2–4 hours	45.50	(25.54)	931	0.01	0.650
5–6 hours	54.36	(29.20)	904	0.10	0.001
>6 hours	59.27	(30.27)	1058	0.11	0.001
**Satisfaction with academic study under present circumstances**					
Yes	39.07	(25.41)	1189	*	
No	60.78	(28.10)	1933	0.27	<0.001
**Smoking**					
No	50.51	(27.60)	2535	*	
Yes	61.14	(33.44)	587	0.15	<0.001

Note:

SD = standard deviation

*Reference category

^†^standardized regression coefficient from the linear regression model

## Discussion

This study is the first measuring the mental health conditions of university students nationwide in Bangladesh early in the COVID-19 outbreak. Prevalence estimates of DAS were, respectively, 76.1%, 71.5% and 70.1% for at least mild symptoms, 62.9%, 63.6% and 58.6% for at least moderate symptoms, 35.2%, 40.3% and 37.7% for at least severe symptoms and 19.7%, 27.5% and 16.5% for at least severe symptoms. According to regression analysis and using a Bonferroni correction for multiple analyses, DASS-21 scores were significantly associated with being female, living in an urban area, not engaging in physical exercise, having dissatisfaction with sleep, browsing the internet for many hours a day (5–6, 6 or more), having dissatisfaction with their pursuit of their academic studies while living under COVID-19 restrictions, and smoking. Several additional measures (being older (25–29 years), having ≥5 family members) were significant at p<0.05 but did not withstand correction for multiple comparisons. Implications are discussed below.

The COVID-19 pandemic has affected almost every country of the world and most countries have introduced lockdown measures [2]. Like in other countries, the Bangladesh government also closed educational institutions [8]. Mental health problems in students are considered a public health challenge [37]. Prior public health emergencies have impacted university students, resulting in students experiencing anxiety, fear, and stress [13].

To the best of our knowledge, the study is the first to examine DAS among university students in a country impacted by quarantines and university closure relating to the COVID-19 pandemic. Although university students are considered an important source of academic capital in Bangladesh as a low-income country, their mental health to date has not been evaluated during the pandemic. Available research has rather often focused on characteristics of the virus [38, 39] and its infectious epidemiology [40–43]. Although a similar mental health study was conducted in China [13, 44], the focus was on the general population.

### Comparison with previous work

Given very large differences between surveys attributable to population under examination, instruments and cultural context, we compared the results with previous work that (i) focused on university students, (ii) made use of the DASS-21 or comparable instruments, with multiple severity levels, and (iii) was conducted in Bangladesh and comparable Asian countries.

**Fig 1:**
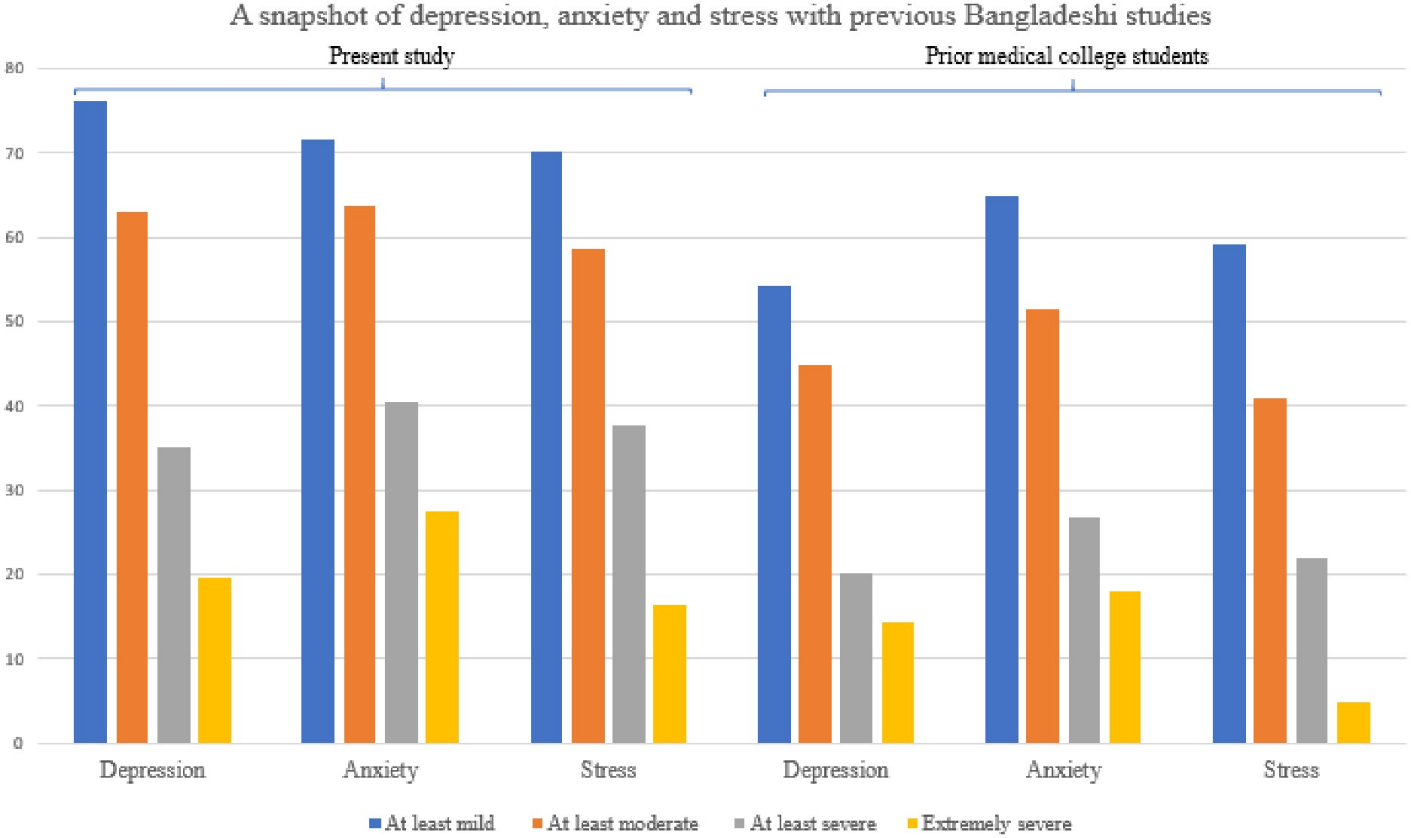
Comparison with previous Bangladeshi student samples. References: Prior studies in medical college students [46].

The current results revealed that many students experienced moderate to extremely severe depression (62.9%), anxiety (63.6%), and stress (58.6%). DAS were highly overlapping, consistent with prior studies of their co-occurrences [45]. The rates of moderate to severe depression were 8.6‬% higher in the current sample than in pre-COVID-19 study among medical college students in Bangladesh (62.9% vs. 54.3%; [46]) but anxiety (64.8%) and stress (59%) were comparable [46] (Fig 1). The prevalence estimates of moderate to extremely severe DAS in the current study were higher compared to a recent study conducted among university students in Bangladesh which reported the prevalence of DAS were 52.2%, 58.1% and 24.9%, respectively [20]. In addition, the levels of moderate to extremely severe depression (62.9% vs. 69.5%) and anxiety (63.1% vs. 61.0%) were comparable to those reported in a recent study (conducted in October 2019, prior to the current study and using Patient Health Questionnaire [PHQ-9] and Generalized Anxiety Disorder [GAD-7] instruments) that included a smaller sample of first-year university students in Bangladesh [21].

Table 4 demonstrates the international work in Asian jurisdictions using comparable methodologies. Prevalence rates of moderate to extremely severe DAS were typically higher in the current study compared to most other previous studies concerning university students. The prevalence of mental health problems may be particularly high because of uncertainty in exams, classes, reopening of the university, and strict social isolation [47], although it may reflect other factors including present Bangladeshi circumstances under COVID-19 (e.g., strict lockdown, home quarantine, spatial distancing). Particular attention is required to attend to mental health concerns in this vulnerable population, along with increasing awareness of factors contributing to the risk of mental health problems.

**Table 4:** Moderate to severe depression, anxiety and stress among university students in Asian jurisdictions.

References	Countries	Depression	Anxiety	Stress
[48]	India	32.0	43.8	43.8
[49]	Pakistan	48.4	73.4	47.6
[27]	Malaysia	37.2	63.0	23.7
[50]	Saudi Arabia	43.0	63.0	41.0
[37]	Turkey	27.1	47.1	27.0
[22]	Egypt	60.0	64.3	62.4

The results of this study showed that the DASS-21 scores were comparatively higher in females than males which is in line with epidemiological research but differs from previous studies in Bangladesh using similar [46] and different instruments [21], that did not report significant gender differences. However, a similar finding (females being more likely to have DAS) was reported in a previous study globally [50]. Moreover, women are more likely to be tasked with additional caretaking duties during the pandemic, which could account for increased levels of symptoms.

A significant association between older age (i.e., 25–29 years) and DASS scores emerged in the current results which was not in the hypothesized direction and dissimilar with prior Bangladeshi studies [20, 21, 46] reporting no association with age/study years. The present findings also suggest a possible association between the numbers of family members (i.e., ≥5) and DASS-21 scores which was not assessed in previous studies [20, 46] but differs from a prior Bangladeshi study using different instruments [21]. However, both of the current findings should be interpreted cautiously as they did not withstand correction for multiple comparisons and thus may not be robust.

Several factors that are relevant to the student population may impact mental well-being including economic difficulties, debt, familial factors, evictions, job losses and unemployment, social disintegration, and poor quality of life [37, 51]. Our study, however, found no significant associations between monthly family income and DASS-21 scores among students, similar to a previous study [27]. The findings also indicated that relationship status or marital status did not relate to DASS-21 scores which is similar with prior Bangladeshi studies [46] but differs from recent reports concerning university students [22, 51]. Moreover, we found significant associations with urban area which is similar to a previous study [52] but dissimilar with other Bangladeshi studies [20, 46] reporting no association with urban residence.

Sleep contributes importantly to health by helping to maintain the biological rhythm of individuals. The present study revealed similar findings of dissatisfactory sleep with DAS, similar to previous studies using different instruments in the same country [21, 53].

The current study found a significant relationship in DAS, and lack of physical exercise as well as excessive internet use, similar to prior reports [20, 21]. A previous study revealed positive associations between problematic internet use and both depression and anxiety [54]. The findings also revealed significant associations with smoking. A previous study in Bangladesh [20] found similar associations between smoking and depression, and globally, a study [50] found similar associations between smoking and DAS. Moreover, people who smoke may be more likely to use tobacco because they cannot be at work during the lockdown period or for other reasons.

### Interpretation

Compared to other studies assessing DAS among university students using the same instrument in the same country, the prevalence estimates of DAS among the university students found in the present study tended to be higher, particularly with respect to stress and depression. These differences may be secondary to the COVID-19 pandemic situation. In addition, the pattern of associations with demographic and potential risk variables showed some differences compared to previous work, which may also be related to the COVID-19 situation. Thus, the lack of significant gender differences in previous work was now replaced by a female preponderance, which may suggest that women are more susceptible to the stresses associated with lockdown and social isolation. Also, the protective effect of being in a relationship identified in previous work did not materialize here, suggesting that this protective effect may be neutralized in the COVID-19-related environment. An association of DAS with older (25–29 years) rather than younger (18–24 years) age during young/emerging adulthood was found; however, the finding did not survive Bonferroni correction for multiple comparisons, contrary to the hypothesis. It is possible that older age in the current COVID-19 circumstances was associated with more responsibilities and financial burden, although this is notion is currently speculative. Replication of these findings is required before more definitive conclusions can be drawn. Longitudinal analyses are required to establish temporality of associations.

### Limitations

This study is not without limitations which should be considered when interpreting the data. First, the study was of a cross-sectional nature so that causality cannot be established. A longitudinal analysis would help in this regard. Second, the study used a self-report approach which may have social desirability and declarative memory biases. Third, the study was conducted in Bangladesh; hence, results may not generalize to other student populations.

## Conclusions

The world is facing serious public health concerns during the COVID-19 pandemic. We gathered new information on DAS among university students during the pandemic. The results may help in identifying mental health concerns and individuals who may be at elevated risk and benefit from interventions. Future longitudinal studies are needed to investigate more closely relationships between mental health problems and COVID-19-related factors and to translate this information into better student health. As during the quarantine many students spend time on the internet (including on social media), online counseling, campaigns, and other awareness programs that may be helpful to reduce the impact of COVID-19 on mental health in this population.

## Supporting information

S1 File Data set- psychological responses during the COVID-19 outbreak among university students in Bangladesh. (XLSX)
